# Early Milk Total and Differential Cell Counts as a Diagnostic Tool to Improve Antimicrobial Therapy Protocols

**DOI:** 10.3390/ani13071143

**Published:** 2023-03-24

**Authors:** Alfonso Zecconi, Francesca Zaghen, Gabriele Meroni, Valerio Sora, Piera Anna Martino, Giulia Laterza, Lucio Zanini

**Affiliations:** 1 Department of Biomedical, Surgical and Dental Sciences, School of Medicine, University of Milano, Via Pascal 36, 20133 Milan, Italy; 2 Department of Clinical and Community Sciences, School of Medicine, University of Milan, Via Celoria 22, 20133 Milan, Italy; 3 Associazione Regionale Allevatori Lombardia, Via Kennedy 30, 26013 Crema, Italy

**Keywords:** mastitis, antimicrobial resistance, One Health, early diagnosis, differential cell count, dry-cow therapy

## Abstract

**Simple Summary:**

The prevention and control of mastitis, from a One Health perspective, should balance the risk of intramammary infections, impairing cow health and welfare, and the need of a reduction of antimicrobial usage (e.g., applying selective dry-cow therapy), thus decreasing antimicrobial resistance. This study showed that the application of an early diagnosis (5–16 days after calving) based on PLCC (neutrophils + lymphocyte count/mL) has good accuracy, a sustainable cost, and could potentially reduce the frequency of antimicrobial treatment compared with conventional diagnostic protocols based on SCC at the first milk test after calving.

**Abstract:**

Mastitis is a major cause of antimicrobial treatments either during lactation or at drying off. From a One Health perspective, there should be a balance between the risk of IMI that may impair cow health and welfare and the reduction of antimicrobial usage to decrease antimicrobial resistance, as may happen when applying selective dry-cow therapy. This reduction may be achieved by an early and accurate diagnosis followed by prudent and rationale therapeutical protocols. This study aims to assess the accuracy of PLCC (neutrophils + lymphocyte count/mL) in identifying cows at risk of having IMI due to major pathogens (*S.aureus*, *Str.agalactiae*, *Str.uberis*, and *Str.dysgalactiae*), and to simulate the impact of this early diagnosis on the potential number of treatments using a decision-tree model. The results of this study showed that PLCC had an overall accuracy of 77.6%. The results of the decision-tree model based on data from the 12 participating herds, with an overall prevalence of major pathogens of 1.5%, showed a potential decrease in the number of treatments of about 30% (from 3.4% to 2.5%) when PLCC in early lactation (days 5–16) was used to identify cows at risk for major pathogens compared with using SCC at the first milk test (days 17–43). The study confirmed that it is possible to improve animal health and reduce the risk of antimicrobial use through early IMI detection based on PLCC and applying a rationale and prudent antimicrobial protocol.

## 1. Introduction

Mastitis is one of the costliest diseases in dairy herds due to direct and indirect costs [1,2,3,4,5]. Moreover, it is one of the major causes of antimicrobial treatments either during lactation or at drying off [6,7]. The importance of reducing antimicrobial treatments to reduce the risk of antimicrobial resistance is well known, and it must not be underestimated also in dairy herds [8,9,10]. Such a decrease should be obtained either by a reduction of clinical and subclinical mastitis cases (prevention) or by an increase in cure rates (efficacy). An increased cure rate is attainable through an early and accurate diagnosis followed by prudent and rationale therapeutical protocols [11,12,13,14].

**Figure 1:**
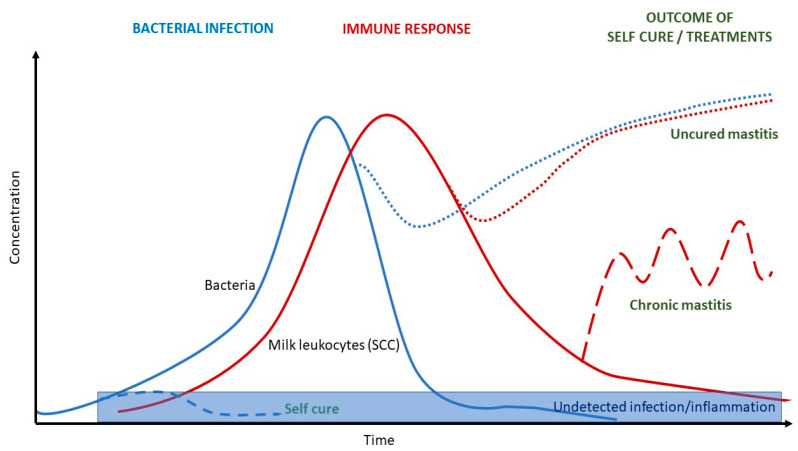
Dynamics of intramammary bacterial infection and udder inflammatory response. The figure models the concentration trend (Y axis) over time of mastitis pathogens and of the leukocytes, hypothesizing different outcomes (cured, uncured, chromicized mastitis), from [15].

Indeed, mastitis is a dynamic pathological process, as summarized in Figure 1 [15]. The teat exposure to pathogens may lead to an infection, and consequently to an inflammatory reaction, usually measured by the means of total and differential cell count [16,17,18]. The inflammatory response as well as the increase and changes in leukocyte responses are detectable with some delay since the infection, and this delay is affected both by cow immune capabilities and by pathogen characteristics [19,20,21]. The microbiological analysis of quarter milk samples is considered the gold standard to identify udder infections [15,22]. However, a surveillance system based on routine microbiological analysis is not practically feasible due to the high costs (analysis + labor) of this approach.

Since the 1970s, the alternative approach applied worldwide is based on the somatic cell count (SCC), which identifies the presence of subclinical mastitis [23,24]. This approach, when applied routinely on a monthly basis (i.e., dairy herd improvement -DHI- milk test), helps to identify the presence of subclinical mastitis, even if the time interval doesn’t guarantee to identify all the cases and, generally, cannot be considered an early diagnosis.

Recently, the application of differential cell count (DSCC) increased the accuracy of subclinical mastitis diagnosis when applied to monthly milk testing [17,25], and it has also shown to be a useful tool to perform an early assessment of the risk of having intramammary infections (IMI) due to major pathogens when applied to quarter milk samples [16].

These evidences suggested we investigate the accuracy of an early test applying SCC and DSCC after calving prior to routine DHI milk test compared to a routine DHI milk test in detecting cows at risk to have IMI caused by major pathogens (*S.aureus*, *Str.agalactiae*, *Str.uberis*, and *Str.dysgalactiae*). These pathogens are the most probable ones requiring an early antimicrobial treatment since they are contagious and diffusive (*S.aureus*, *Str.agalactiae*) or cause clinical mastitis (*Str.uberis*, and *Str.dysgalactiae*). An early treatment of these bacteria showed to increase the cure rate, thus avoiding the development of antimicrobial resistance related to non-efficacious treatments in later lactation or in presence of a clinical outcome [19].

Decision tree models are increasingly applied in human and medical diagnostic protocols, as well as in other scientific areas [26]. They are considered transparent, allowing the decision-maker to examine and understand the decision model. In addition, each path in the decision tree can be regarded as a decision rule. Decision tree models describe events in chronological order and include all the quantitative data relevant to the problem. They use nodes and branches connecting the nodes to show graphically how decisions unfold through time.

The aim of this study was to estimate and compare the effects on the potential number of antimicrobial treatments obtained by the application of a decision model approach for early detection of cows at risk for major pathogens based on three different cellular markers (SCC, DSCC, and the combination of the two -PLCC).

## 2. Materials and Methods

### 2.1. Herd and Cow Selection

This study considered about 3084 cows that calved in the period of January 2019–December 2021 from 12 dairy herds in the Lombardy Region and that are enrolled in the Italian Breeder Association (AIA) monthly individual DHI milk test. The selected herds should also take quarter milk samples after calving as a routine procedure to identify IMI as early as possible. The herd size was in the range 90–500 lactating cows, and 95% of the cows were Italian Friesian.

### 2.2. Sample Collection and Analysis

Individual cow samplings were performed by certified methods currently applied by AIA at the laboratories of the Regional Breeders Association of Lombardy (ARAL) by the means of Lactocorder™ (WMB AG, Balgach, Switzerland). Samples were taken every 4–5 weeks, as required by the milk test procedure, delivered refrigerated to ARAL labs the same day, and analyzed within 30 h of sampling.

Milk analyses included SCC and DSCC and were carried out on Fossomatic™ 7DC (Foss A/S, Hillerød, Denmark). The DSCC was assessed by the method described by Damm et al. [27]. This method allows to identify within a milk sample the macrophages (MAC) and the combination of polymorphonuclear leukocytes (PMN) and lymphocytes (LYM). DSCC is expressed as the combined proportion (%) of PMN and LYM in the overall count of milk cells.

Quarter milk samples (QMS) were taken following the procedure described by N.M.C., 2017 [28] by the milkers, delivered refrigerated to ARAL laboratories where bacteriological analyses were performed by streaking 0.01 mL of QMS on blood agar plate with 5% (*v*/*v*) bovine blood according to N.M.C., 2017 [28]. After incubation (18–24 h at 37 °C), the colonies recovered were identified by Vitek™ system (Biomerieux, Lion, France). All the samples were taken at least five d after calving to minimize the influence of colostrum, as suggested [24,29]. These were samples taken routinely as requested by the herd health management protocol, which aimed to identify cows with IMI after calving.

Based on the results of the bacteriological analysis, a quarter was classified similarly to a previous paper [12]. Briefly, a quarter of the samples were classified as positive for an IMI due to major pathogens (MaJP) when 1 or more colonies of *Str.agalactiae*, and *S.aureus* were isolated, or when 5 or more colonies of *Str.uberis* and *Str.dysgalactiae* were isolated. It was considered positive for an IMI due to other bacteria when 5 or more colonies of the same species of Gram-negative pathogens (*E. coli*, *Klebsiella* spp., other coliforms) were isolated, or when 10 or more colonies of the same genus (coagulase negative *Staphylococcus* species, other environmental *Streptococcus* species, *Enterococcus* species) were isolated. It was classified as negative when no colonies were recovered, or their number was below the values defined in the previous classes and contaminated when ≥3 different colony types were isolated from the milk sample.

This classification is based on the pathogenic characteristics of the bacteria, and the usefulness of the application of antimicrobial therapy under the principle of a prudent application of antimicrobials. Therefore, the antimicrobial therapy should be restricted to cows infected with bacteria that have very high chances to induce clinical mastitis and/or spread contagious IMI, which can (cost-) effectively be controlled by antimicrobial treatment [25,26,27].

### 2.3. Statistical Analysis

Only cows with a quarter milk sample within ±3 days of the first individual milk test were considered. The data were collected in a database, including herdID, cowID, days in milk (DIM), results of the bacteriological analysis by quarter, SCC and DSCC from the DHI milk test, and a variable (PLCC) calculated by multiplying SCC×DSCC. This variable represents the total number of PMN + LYM/mL.

The identification of the optimal cut-off values to identify cows at risk for IMI by the markers considered (SCC, DSCC, and PLCC), and the accuracy of the diagnosis were obtained by calculation of ROC curves (Xlstat 2022.5.1; Addinsoft, New York, NY, USA). The response variable considered (disease) was defined as positive when an IMI due to MajP (*Str.agalactiae*, *Str.uberis*, *Str.dysgalactiae*, and *S.aureus*) was recorded in at least one quarter. It was considered negative when none of the quarters had an IMI due to MajP. The analysis allows to calculate for each cut-off point the following variables: sensitivity (Se), specificity (Sp), positive predictive value (PPV), negative predictive value (NPV), and diagnostic accuracy (effectiveness), expressed as a proportion of correctly classified subjects (True positive TP + True negative TN) among all subjects (TP + TN + False positive FP + False Negative FN).

### 2.4. Decision Tree Analysis

**Figure 2:**
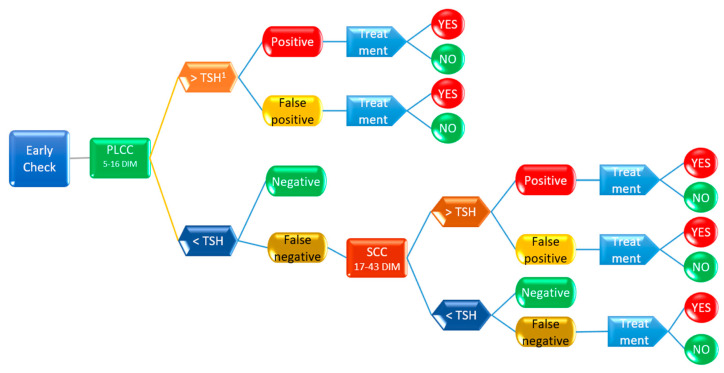
Summary of the decision tree model calculating the risk for intramammary treatment based on PLCC in the A period (5–16 days from calving). All the potential outcomes were considered for each of the nodes (positive, false positive, or negative), leading to the chances of a treatment. See Supplementary Figure S1 for the probability of each outcome. ^1^ TSH: threshold specific for each marker.

**Figure 3:**
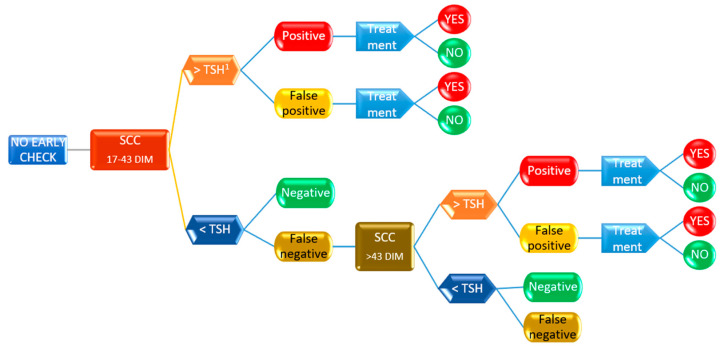
Summary of the decision tree model calculating the risk for intramammary treatment based on SCC in the B period (17–43 days from calving). All the potential outcomes were considered for each of the nodes (positive, false positive, or negative), leading to the chances of a treatment. See Supplementary Figure S2 for the probability of each outcome. ^1^ TSH: threshold specific for each marker.

Decision tree models describe events in chronological order and use nodes and branches connecting the nodes to show graphically how decisions unfold through time. A decision node (hexagons in Figure 2 and Figure 3) is a time when a decision must be made. A chance node (rounded rectangles) is a time when an uncertain outcome is revealed. An end node (blue arrows) indicates that the problem is over, and all decisions have been made (circles). A probability is associated with each of the decisions (circles). Branches connect nodes, and there are the following types of branches in the model: a branch extending from a decision node for every possible decision at that point in time; and branches for each possible outcome of a change node.

The value of an end node is the sum of the values of each branch in the path from the root. The percentage (likelihood) of an end node is the product of each branch in the path from the root. Working from right to left, the value of a chance node is the weighted average of the values of the nodes that that chance node branches to, and the value of a decision node is the value of the node on the branch of that decision node.

The decision-tree analysis was performed by the means of PrecisionTree™ software (ver. 8.3.2. 2022 Palisade corp., Ithaca, NY, USA) to assess the probability of a treatment, following two different diagnostic paths: the first one applying PLCC in the period 5–16 DIM, followed by QMS on cows at risk (PLCC > then calculated threshold) to confirm the presence of MajP; and the second one based on SCC at the first routine DHI (17–43 DIM) also followed by microbiological analysis of QMS. The software determines the best decision to make at each decision node [16]. The decision tree model is summarized in Figure 2 and Figure 3, while the complete model, including the probability values for each node, is reported in Supplementary Figures S1 and S2.

## 3. Results

### 3.1. Data Description

The original dataset was checked to identify missing data, such as the absence of SCC or DSCC, and for bacteriologically contaminated samples. All the records with these features were discarded, and the final database included 2733 valid records. These records are classified into three subsets based on the days in milk when the milk test record (MTR) was performed: A (5–16 d), B (17–43 d) and C (44–300 d). The MTR data were summarized in Table 1. The mean values observed for all the cellular variables (SCC, DSCC, and PLCC) are very close. However, the C group showed highest mean values in for all the markers when compared with the other subsets, while the lowest means were observed in the B subset, except for DSCC.

Table 2 reports the distribution of IMI on a quarter basis, classified into the three lactational periods. The prevalence of MajP was very low both in the A and B subsets, while other pathogens showed to have a high prevalence throughout the lactation, with the lowest value in the B subset, where the highest proportion of negative results was found. Overall, *S.aureus* represented 45% of the MajP isolated, while *Str.uberis*, *Str.dysgalactiae* and *Str.agalactiae* represented, respectively, 32%, 22%, and 1% of the isolates.

### 3.2. SCC, DSCC and PLCC as Markers for Major Pathogens

The results of ROC analysis to identify the thresholds for the three cellular markers with the highest accuracy in identifying cows at risk for major pathogens IMI are reported in Table 3. As expected, the thresholds were different for each marker among the different lactation periods considered. The highest accuracy values were observed in the A subset for all of the three markers. PLCC showed the highest accuracy among the markers in all the lactational period except for the C subset. The sensitivity was higher in the C subset compared to the other periods, while the highest specificity values were observed in the A period.

### 3.3. Decsion Tree Model for Antimicrobial Treatment Probability

The previous results support the hypothesis that PLCC measured in the very early phase of lactation is a tool to identify cows at risk of having an IMI due to MajP [12]. This positive aspect should also be complemented by an equal or reduced use of antimicrobials, when compared with a deferred diagnosis. Therefore, before suggesting it in practice, we simulate two different approaches to assessing the probability of antimicrobial treatments. The first one included early detection with PLCC measurement, and the alternative, representing a conventional approach, based on SCC obtained at the first DHI milk test to identify cows at risk.

The thresholds for decision nodes (PLCC and SCC) and the values for test performances (proportion of respectively true positive, false positive, true negative, and false negative) after chance nodes were presented in Table 3, and the model applied was reported in Figure 2, Figure 3, Figures S1 and S2. The probability of treatment was fixed at 90% for true positive cases and 10% for false positive and false negative cases. This latter probability was included to take care of any potential bias that could occur in practice.

The results of the statistical analysis showed that the number of treatments with the early approach was estimated at 2.5% and 2.1%, respectively, for true positive and false positive samples, while it was 3.4% and 5.6% for true positive and false positive samples, applying the conventional SCC approach at the first DHI milk test. Therefore, we estimate that the early diagnostic approach proposed led to a decrease of nearly 50% in the potential treatment frequency (4.6% vs. 9%).

## 4. Discussion

The constant improvement of dairy herd management led to a decrease in SCC in many herds worldwide [5,30], and the average SCC values for the herds included in this study support these observations since they do not exceed 125,000 cells/mL (log_10_ 5.1).

The decrease in average SCC is also due to the use of blanket dry-cow therapy, which was very common in most of the dairy herds worldwide until recently. However, the concerns about antimicrobial resistance (AMR) spreading suggest that applying more prudent approaches and reducing the use of antimicrobials in food producing animals. Among these prudent protocols, the selective dry-cow therapy is one of the most promising, and it is now compulsory within the European Union (EU Regulation 2019/6). The increasing application of selective antimicrobial therapy at drying-off is decreasing the use of antimicrobials, but as a relatively large proportion of cows are not treated with antimicrobial at drying-off, thus increasing, this could potentially lead to an increase in new IMI after calving [31,32]. These IMI are an important source of clinical mastitis [33]; therefore, they may be treated as early as possible to avoid this occurrence. The same concerns about AMR and the need to increase the sustainability of dairy herds suggest also to restricting the use of antimicrobials to cows infected with bacteria that can be effectively controlled by antimicrobial treatment and are a source of a significant decrease in milk yield [27,28].

From a One Health perspective, there should be a balance between the risk of IMI that may impair cow health and welfare and the reduction of antimicrobial usage, leading to a reduction in AMR. One of the possible ways to achieve this balance is identifying IMI after calving due to major pathogens, and treat them, when necessary, to avoid the development of clinical or chronical mastitis that would also impair dairy herd sustainability [34].

The most effective way to identify the infected cows is to perform a microbiological analysis of quarter milk samples within two weeks after calving, as confirmed by the positive results of contagious control programs [14,35]. However, QMS microbiological analysis may be expensive, and farmers are reluctant to apply routinely this procedure also for the labor required. Early treatments of positive cows are generally considered to have a high cure rate, leading to stronger and quicker restoration of milk production both in terms of quality and quantity [11].

Therefore, new approaches should be developed characterized by low cost and ease of implementation during the milking routine. One of the most promising approaches with these features showed up to be individual milk sampling and the assessment of both SCC and DSCC, and the calculation of PLCC (number of PMNs and lymphocytes/mL) [16]. The results of this study confirm previous ones [16] with an overall accuracy, for the three markers in the range of 73.6–77.6% (Table 3), with the highest value (77.6%) for PLCC. Moreover, PLCC measured in the first 16 days after calving showed a higher accuracy and a higher specificity than SCC in the same period of lactation, and even higher in the period 17–43 DIM. Since most of the cows were not infected by MajP, the higher specificity results in a lower number of false positive cows, thus reducing the number of microbiological analyses and the related costs. The SCC and DSCC measurements are performed on the same milk sample and, in Lombardy, without difference in cost.

The diagnostic approach presented in this study could be included in a decisional process involving early sampling and diagnosis to identify cows at risk through PLCC calculation, the microbiological analysis of quarter milk samples from cows at risk (PLCC higher than threshold) to confirm the presence of major pathogens, and the treatment of infected quarters, when suggested by the protocols developed by the herd veterinarian. The practical advantage of applying this protocol in comparison with the most common approach to checking cows that are over a threshold of 200,000 cell/mL at the first DHI sample available (generally after 15 DIM) is that it allows for sustainable early diagnosis with higher accuracy, thus allowing to identify cow at risk of developing clinical mastitis or to spreading the infection. Moreover, this approach should also lead to an overall decrease in antimicrobial treatments, obtained by the reduction of the spread of infections (in the case of contagious pathogens) and the reduction of clinical case incidence (in the case of environmental pathogens).

To our knowledge, there are no data from practice supporting this diagnostic and therapeutic approach yet. Moreover, this approach may raise concerns about on the potential increase in antimicrobial treatments. To gather information on this latter aspect, we apply a decision-tree model simulating the potential impact of treatments applying early detection (5–16 DIM) based on the PLCC values (threshold 163,500 cells/mL) vs. a conventional approach based on evaluation of SCC (threshold 40,000 cells/mL) at the first DHI milk test (17–43 DIM), as usually done in many herds. The simulation model, based on the data from 12 different herds with a prevalence of MajP of 1.5% in both periods A and B, allows to estimate the potential impact of this approach before the application under field conditions.

The results of the decision-tree model showed that the analysis of PLCC in the A period decreased potential treatments by about 30% compared to the B period approach (2.5% vs. 3.4%). The reduction was also consistent when false positive samples were considered (with a supposed treatment rate of 10%). Indeed, the reduction was by 63% (2.1% vs. 5.6%). It should be noticed that the increase of treatment was not due to a change in the prevalence of IMI since they the values were the same either in the A and in the B period (Table 2), but to the higher value of specificity when PLCC is applied vs. SCC, thus reducing the false positive frequency.

The thresholds applied were calculated in cows mainly of the Italian Friesian breed, and there is evidence that breed may affect DSCC and SCC values [32]; however, the low values of the thresholds for both SCC and PLCC and their measurement in the first 45 DIM, in our opinion, make them suitable also for the other breeds, even if characterized by different mean values.

Moreover, these results were obtained applying the optimal threshold calculated for SCC (40,000 cells/mL), which is quite lower than the one usually applied (200,000 cells/mL) [17,24]. Applying this latter value, the sensitivity would decrease to 49%, instead of 88%, with a large proportion of infected animals going undetected.

These results support the application under field condition of the diagnostic and therapeutical protocol and their evaluation in practice. The positive results obtained in well managed herds with a low prevalence of MajP suggest that such an approach would be even more profitable in herds with higher prevalence of MajP, would lead to even larger risks since it may be hypothesized that the higher prevalence is associated with a lower management level.

## 5. Conclusions

A One Health approach applied to dairy herd management requires that the improvement in human health (i.e., decrease of AMR) should not be impaired by a decrease in animal (cow) health. The limits in applying blanket dry-cow therapy may increase the risks of new IMI leading to clinical and chronic mastitis, which is the opposite of the aims of a One Health approach. The availability of new technologies (DSCC) and the development of new diagnostic approaches, shows as it is possible to apply innovative early IMI detection protocols under field conditions. Despite neither of the three markers considered achieved the accuracy of microbiological analysis in detecting IMI, PLCC showed the best results compared to SCC and DSCC. The result of this study shows that it is potentially possible to achieve a win-win situation by increasing human and animal health through an early IMI detection based on the PMN + LYM count/mL (PLCC) and applying a rationale and prudent antimicrobial protocol.

## Figures and Tables

**Table 1:** Description of sample characteristics: mean values ± standard deviation of cow days in milk and cellular markers within the three lactational periods considered.

Lactation Period	N	Days in Milk ± Std.dev (d)	SCC ^1^ ± Std.dev (Log_10_/mL)	DSCC ^2^ ± Std.dev (%)	PLCC ^3^ ± Std.dev (Log_10_/mL)
A (5–16 d)	995	11.0 ± 3.3	5.0 ± 0.6	63.5 ± 15.9	4.8 ± 0.7
B (17–43 d)	831	24.9 ± 7.00	4.9 ± 0.7	64.0 ± 18.3	4.7 ± 0.7
C (44–300 d)	907	167.7 ± 93.2	5.1 ± 0.6	64.2 ± 17.6	4.9 ± 0.7

^1^ SCC = somatic cell count. ^2^ DSCC = differential cell count. ^3^ PLCC = SCC × DSCC.

**Table 2:** Mammary gland health status: distribution of intramammary infections based on quarter milk sampling within the three lactational periods considered.

Lactation Period	Quarter (N)	MajP ^1^	Other Pathogens	Negative
A (5–16 d)	2407	1.5%	38.0%	60.5%
B (17–43 d)	2336	1.6%	28.2%	70.3%
C (44–300 d)	2001	6.8%	38.1%	55.2%

^1^ MajP: major pathogens *Str.agalactiae*, *Str.uberis*, *Str.dysgalactiae*, and *S.aureus*.

**Table 3:** Results of ROC analysis to identify the thresholds for the three cellular markers with the highest accuracy in identifying MajP classified for the three lactational periods considered.

Lactation Period	Parameter	Thres Hold	Sensitivity	Lower Bound (95%)	Upper Bound (95%)	Specificity	Lower Bound (95%)	Upper Bound (95%)	PPV ^4^	NPV ^5^	Accuracy
A (5–16 d)	PLCC ^1^	163,500	0.700	0.544	0.819	0.779	0.752	0.804	0.117	0.984	0.776
	SCC ^2^	198,000	0.725	0.570	0.839	0.756	0.728	0.782	0.111	0.985	0.755
	DSCC ^3^	75.2%	0.575	0.422	0.715	0.742	0.714	0.769	0.086	0.977	0.736
B (17–43 d)	PLCC	53,000	0.686	0.519	0.815	0.613	0.579	0.646	0.072	0.978	0.616
	SCC	40,000	0.886	0.733	0.959	0.418	0.385	0.453	0.063	0.988	0.438
	DSCC	68.7%	0.686	0.519	0.815	0.563	0.528	0.597	0.065	0.976	0.568
C (44–300 d)	PLCC	84,000	0.821	0.749	0.876	0.610	0.575	0.644	0.278	0.949	0.643
	SCC	140,000	0.800	0.725	0.858	0.635	0.600	0.668	0.286	0.946	0.660
	DSCC	74.4%	0.586	0.503	0.664	0.729	0.696	0.759	0.283	0.906	0.707

^1^ PLCC = SCC × DSCC; ^2^ SCC = somatic cell count; ^3^ DSCC = differential cell count. ^4^ PPV = positive predictive value; ^5^ NPV = negative predictive value.

## Data Availability

Restrictions apply to the availability of these data. Data was obtained from Associazione Regionale Allevatori della Lombardia and are available from the authors with the permission of Associazione Regionale Allevatori della Lombardia (a.ferla@aral.lom.it).

## Supplementary Materials

The following supporting information can be downloaded at: https://www.mdpi.com/article/10.3390/ani13071143/s1, Figure S1: decision tree model calculating the risk for intramammary treatment based on PLCC in the A period (5–16 days from calving). All the potential outcomes were considered for each of the nodes (positive, false positive, negative), leading to the probability of a treatment; Figure S2: decision tree model calculating the risk for intramammary treatment based on SCC in the B period (17–43 days from calving). All the potential outcomes were considered for each of the nodes (positive, false positive, negative), leading to the chances of a treatment.
