# Supplementary figures and images for: Early Milk Total and Differential Cell Counts as a Diagnostic Tool to Improve Antimicrobial Therapy Protocols
Source: Animals (Basel). 2023 Mar 24;13(7):1143. doi: 10.3390/ani13071143 (PMC10093194; doi:10.3390/ani13071143)

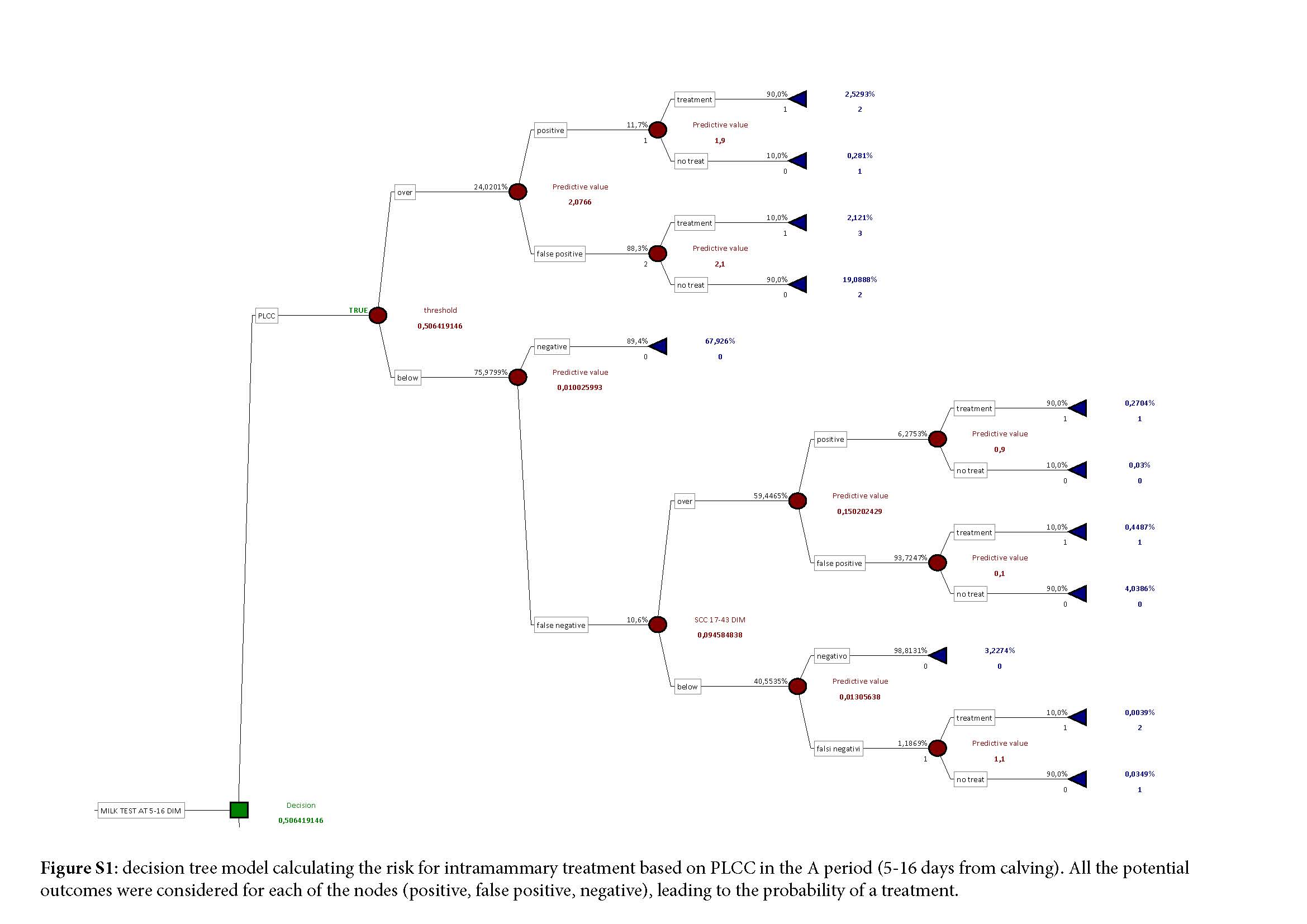

Supplement: Supplementary file 1 [file animals-13-01143-s001.zip › figure 1.jpg]

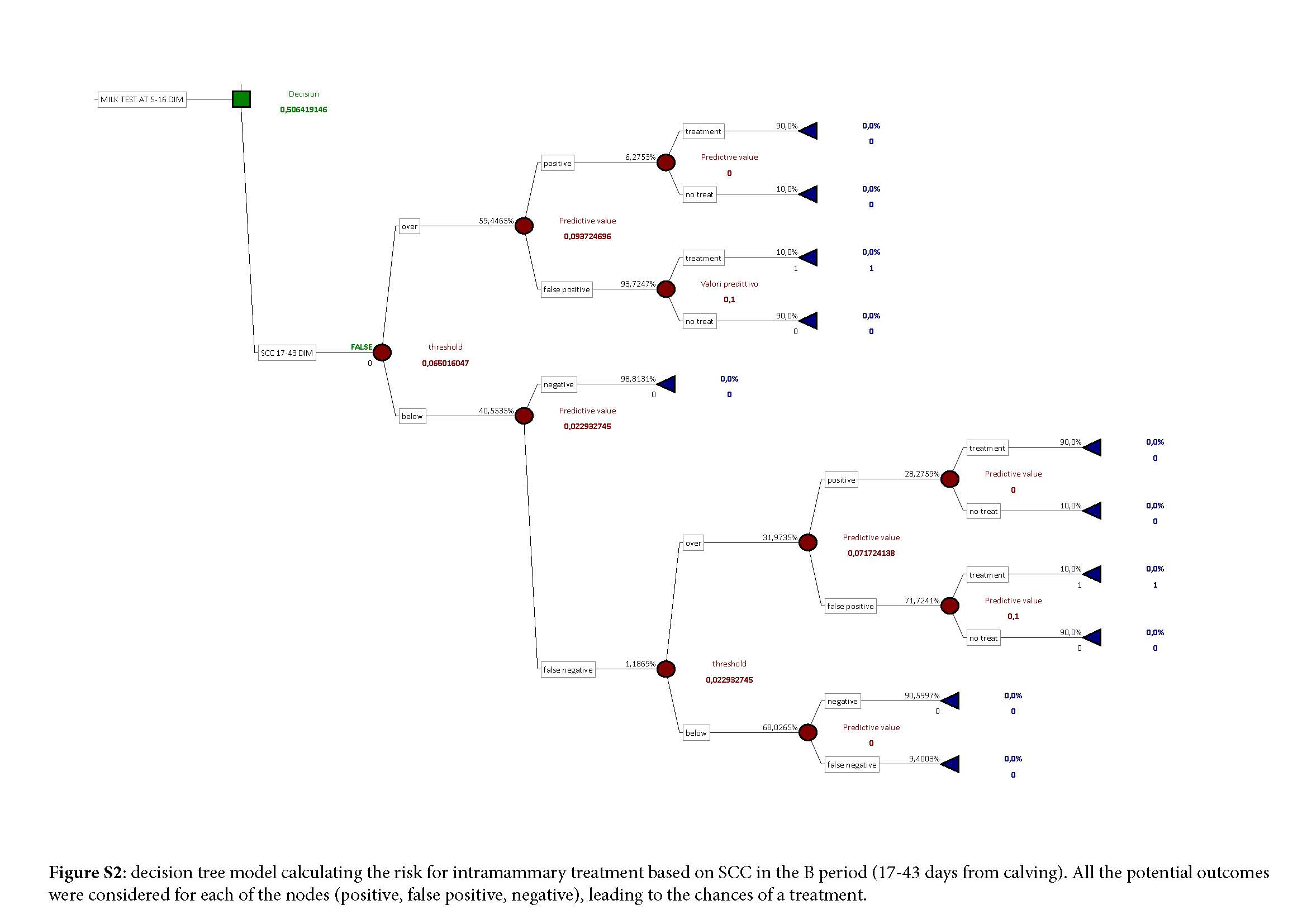

Supplement: Supplementary file 1 [file animals-13-01143-s001.zip › Figure 2.jpg]
